# Celestial Object Imaging Model and Parameter Optimization for an Optical Navigation Sensor Based on the Well Capacity Adjusting Scheme

**DOI:** 10.3390/s17040915

**Published:** 2017-04-21

**Authors:** Hao Wang, Jie Jiang, Guangjun Zhang

**Affiliations:** Key Laboratory of Precision Opto-mechatronics Technology, Ministry of Education, School of Instrumentation Science and Opto-electronics Engineering, Beihang University, No. 37 Xueyuan Road, Haidian District, Beijing 100191, China; topgun_wh@126.com (H.W.); guangjunzhang@buaa.edu.cn (G.Z.)

**Keywords:** optical navigation sensor, well capacity adjusting, star centroid estimation, edge extraction, exposure parameter optimization

## Abstract

The simultaneous extraction of optical navigation measurements from a target celestial body and star images is essential for autonomous optical navigation. Generally, a single optical navigation sensor cannot simultaneously image the target celestial body and stars well-exposed because their irradiance difference is generally large. Multi-sensor integration or complex image processing algorithms are commonly utilized to solve the said problem. This study analyzes and demonstrates the feasibility of simultaneously imaging the target celestial body and stars well-exposed within a single exposure through a single field of view (FOV) optical navigation sensor using the well capacity adjusting (WCA) scheme. First, the irradiance characteristics of the celestial body are analyzed. Then, the celestial body edge model and star spot imaging model are established when the WCA scheme is applied. Furthermore, the effect of exposure parameters on the accuracy of star centroiding and edge extraction is analyzed using the proposed model. Optimal exposure parameters are also derived by conducting Monte Carlo simulation to obtain the best performance of the navigation sensor. Finally, laboratorial and night sky experiments are performed to validate the correctness of the proposed model and optimal exposure parameters.

## 1. Introduction

Optical autonomous navigation is a key technology in deep space exploration. This process is usually accomplished by multi-sensor integration, such as star sensors, navigation cameras, inertial measurement devices, and other equipment. The attitude information of a spacecraft is obtained by a star sensor and inertial measurement element. The navigation camera captures the target celestial image with background stars and extracts the target celestial line-of-sight (LOS) vector according to the current spacecraft attitude. Then, the spacecraft position can be calculated by integrating these optical navigation measurements according to the geometric relationship. An optical navigation system with multi-sensor integration is not only complicated in structure and has high cost and power consumption but also has installation errors between sensors, which further restricts the improvement of navigation accuracy. The best solution for deep space exploration missions is when a single navigation sensor can simultaneously obtain the attitude and LOS vector from the sensor to the centroid of the target celestial body. This approach requires the sensor to image the stars and target celestial body and to extract their navigation measurements simultaneously. However, a large gap exists between the irradiance of stars and the target celestial body. For reference, standard image sensors have a dynamic range of 40 dB to 70 dB [1], which is insufficient to ensure that the target celestial body and stars are well-exposed simultaneously. The problem of insufficient dynamic range for image sensors can be generally solved in three ways.

The design of optical systems to lower the incident flux of a celestial body is first considered. In Reference [2], the combined Earth-/star sensor for attitude and orbit determination of geostationary satellites is investigated. The combined Earth-/star sensor has two fields of view (FOV) for the observation of the Earth and stars. The two FOVs are combined on the detector through a beamsplitter. The partially transmissive mirror reflects 91% of starlight onto the detector, while transmitting only 9% of the Earth’s brightness. The Earth’s incident flux is further reduced by a filter. This method directly lowers the incident flux of the high irradiance target from the source, which is convenient for the subsequent image processing. A disadvantage of this approach is the complexity of the optical system design, higher weight, and higher costs.

The second method enhances the dynamic range by image processing algorithms. In [3,4,5,6], multi-exposure fusion techniques are adopted to enhance the dynamic range. A set of different exposure images is obtained. Then, these images are fused into an image where all scenes or areas of interest appear well exposed. The advantage of the multi-exposure technique is that it can enhance the dynamic range without degrading the signal-to-noise ratio (SNR) [7]. The main drawback of exposure fusion is its limitation to static scenes, and any object movement incurs severe ghosting artifacts in the fused result. Given that a spacecraft is always in the motion state, this method is inapplicable in such condition.

New image sensor designs are also proposed to attain an extended dynamic range. The photocurrent in logarithmic response image sensors is fed to a resistor with a logarithmic current-voltage characteristic [8,9,10]. Logarithmic-response image sensors can obtain a wide dynamic range, but it has several disadvantages (i.e., image lag, low SNR, large fixed pattern noise, and poor image quality). This undesired lag effect is most pronounced at low light conditions, and it is caused by a long settling time constant that can exceed the frame time. Another wide dynamic image sensor based on time-to-saturation information was reported in [11,12,13,14]. The pixel attains its saturation level and extrapolates the incident light by measuring and recording the time required to attain the saturation state. The light intensity is derived by the information on the time stored in the memory of each pixel and then the final image can be reconstructed. However, each pixel of the detector requires a signal detection circuit, comparator, digital memory, and other components to detect the saturation state and storage time information. This condition results in large pixel sizes and low fill factor, which limit the sensor resolution. The well capacity adjusting (WCA) scheme described by Knight [15] and Sayag [16] and implemented by Decker [17] compresses the sensor’s current versus charge response curve using a lateral overflow gate. This technology is currently widely employed by integrating a lateral overflow integration capacitor in a pixel in complementary metal-oxide-semiconductor (CMOS) detectors [18,19,20]. The well capacity is monotonically increased once or multiple times to its maximum value during integration. The accumulated photoelectrons of the high irradiance signal are suppressed, but the low irradiance signal is unaffected. The WCA scheme enhances the dynamic range, but at the expense of substantial degradation in SNR.

In [21], the navigation camera used a sequence of long and short exposures for optical navigation. A short exposure in which the celestial body is not saturated will permit determination of the celestial body’s location within the image. A long exposure will permit determination of the stars’ location within the image. Setting a long exposure time to ensure that the dim stars satisfy the detection SNR limit is necessary to ensure that the navigation sensor has a reliable attitude determination function. However, a long exposure time can lead to the overexposure of the target celestial body, which results in the expansion of the image shape because the apparent diameter increases and high stray light effect which will overwhelm the natural response to the target stars. Given that the WCA scheme is widely utilized in CMOS image sensors, high dynamic range images can be obtained by a single exposure with this technique.

This study first analyzes the irradiance characteristics of a celestial body. Then, the celestial body edge model and star spot imaging model are established when the WCA scheme is applied. The effect of exposure parameters on the accuracy of star centroid estimation and edge detection is analyzed based on the proposed model. The exposure parameters are optimized to ensure that the optical navigation measurements satisfy the requirement of navigation accuracy. Comparing with the conventional navigation sensors, this study provides a feasible method for the study of a miniaturized single FOV optical navigation sensor, which is less cost, less weight, simple to design and can obtain attitude information and LOS vector from the sensor to the centroid of the target celestial body simultaneously. This navigation sensor has a strong applicability and can be utilized for a variety of navigation tasks.

## 2. Irradiance Characteristics of a Celestial Body

The study of the irradiance characteristics of a celestial body is the prerequisite for the optimization of the exposure parameters of navigation sensors. The irradiance of a celestial body received by the detector is analyzed in this section.

**Figure 1:**
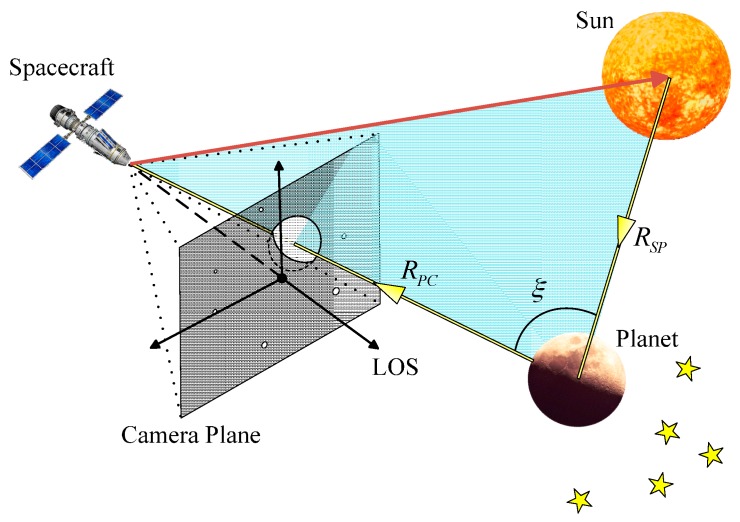
Spatial position relationship between the sensor and target celestial body.

Figure 1 shows the spatial position relationship between the navigation sensor and the observed target. A planet typically emits energy in a manner that reflects solar radiation energy. The irradiance of the sun is assumed to be isotropic because the irradiance is inversely proportional to the square of the distance [22]. Thus, the irradiance received at the target celestial surface is expressed as:

(1) $$I_{P}=I_{sun}{\left(r_{S}/R_{SP}\right)}^{2}$$

where $I_{sun}$ is the irradiance of the sun, $r_{S}$ is the radius of the sun, and $R_{SP}$ is the distance between the sun and the target celestial body. The incident energy on the surface of a planet is only partially reflected back into cosmic space, whereas the rest is absorbed by the surface. $r_{P}$ is defined as the radius of the celestial body, A is the Bond albedo that represents the fraction of energy incident on an astronomical body scattered back into space at all wavelengths and phase angles. Thus, the total radiant flux reflected by the surface of the celestial body is expressed as:

(2) $$L_{R}=\frac{\pi Ar_{P}^{2}r_{S}^{2}}{R_{SP}^{2}}\cdot I_{sun}$$

We consider that navigation sensor observes the planet at a distance of $R_{PC}$. Only a part of the illuminated area of the celestial body can be observed in most cases. If the surface of a planet is assumed to be homogeneous, then the distribution of the reflected radiant energy only depends on two factors, distance $R_{PC}$ and phase angle ξ which is the angle at the celestial body object between the Sun and the observer. Phase function is the ratio of the reflected radiant flux at phase angle ξ to the radiant flux at zero phase angle, which is denoted as P(ξ). When $\xi =0^{{}^{\circ}}$, P(ξ)=1. Thus, the irradiance received by the navigation sensor is expressed in the following form:

(3) $$I_{C}=CP\left(\xi \right)\cdot \frac{L_{R}}{4\pi R_{PC}^{2}}=\frac{CAr_{P}^{2}r_{S}^{2}}{4R_{PC}^{2}R_{SP}^{2}}\cdot P\left(\xi \right)\cdot I_{sun}$$

where C is a constant. The total reflected energy of the celestial body is distributed on a spherical surface. As the distance increases, the radiant flux density decreases, but the total radiant flux remains the same. Therefore, the total radiant flux on the sphere from the center of the celestial body of radius $R_{PC}$ is expressed as:

(4) $$L_{R}=\int_{S}I_{C}dS=\int_{S}\frac{CP\left(\xi \right)L_{R}}{4\pi R_{PC}^{2}}dS$$

**Figure 2:**
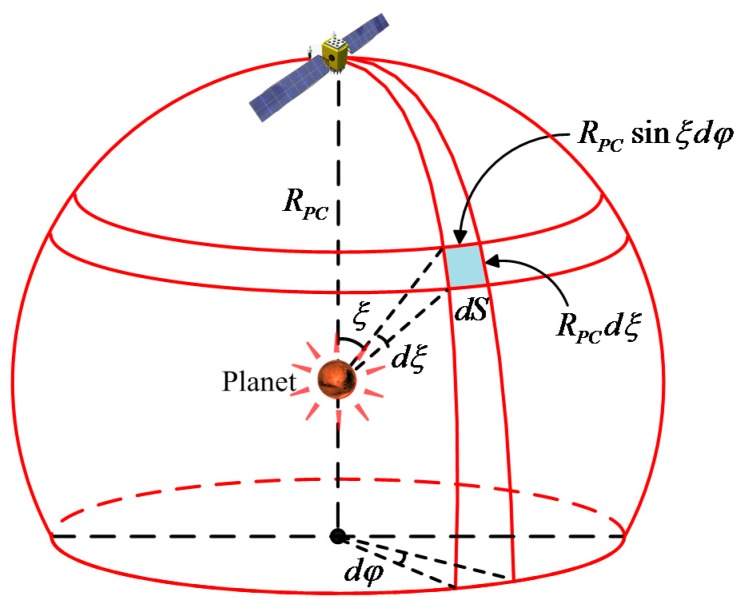
Reflected radiation flux over a sphere.

Figure 2 shows that the area dS can be expressed in terms of its coordinates as $dS=R_{PC}^{2}\sin\xi d\varphi d\xi$. Therefore, C is derived as:

(5) $$C=\frac{4\pi R_{PC}^{2}}{\int_{S}P\left(\xi \right)dS}=\frac{4\pi }{\int_{\varphi =0}^{\varphi =2\pi }\int_{\xi =0}^{\xi =\pi }P\left(\xi \right)\sin\xi d\varphi d\xi }=\frac{2}{\int_{0}^{\pi }P\left(\xi \right)\sin\xi d\xi }$$

In astronomy, the Bond albedo (A) is related to the geometric albedo (ρ) by the expression A=ρq [23], where:

(6) $$q=2\int_{0}^{\pi }\frac{I\left(\xi \right)}{I\left(0\right)}\sin\xi d\xi =2\int_{0}^{\pi }P\left(\xi \right)\sin\xi d\xi$$

Thus, the constant C must obey the following relationship:

(7) $$C=\frac{4\rho }{A}$$

The previously analysis shows that the irradiance received by the sensor can be expressed as:

(8) $$I_{C}=\frac{\rho r_{P}^{2}r_{S}^{2}}{R_{PC}^{2}R_{SP}^{2}}\cdot P\left(\xi \right)\cdot I_{sun}$$

Equation (8) shows that $I_{C}$ is the function of $R_{PC}$, $R_{SP}$, and phase angle ξ. Visual magnitude is the relative quantity generally adopted to measure the irradiance of a celestial object. For example, when the Moon is observed in a geosynchronous orbit, the visual magnitude varies from −2.5 to −12.74. The irradiance of a celestial body is commonly higher than that of stars.

## 3. Celestial Object Imaging Model based on the Well Capacity Adjusting Scheme

**Figure 3:**
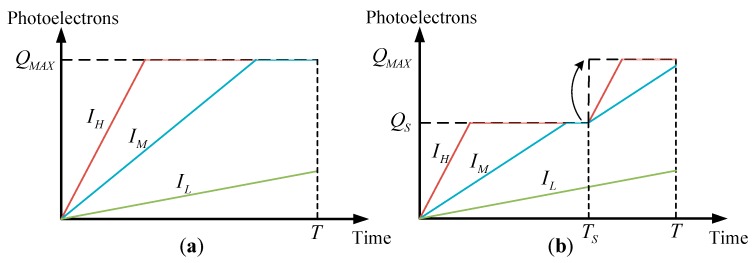
(**a**) Accumulated photoelectrons versus time in normal integration mode; and (**b**) accumulated photoelectrons versus time using the WCA scheme.

The WCA scheme is widely employed in CMOS image sensors. During integration period of the WCA scheme, well capacity of a certain pixel is increased several times to extend the range of incident signal. The dynamic range which is defined as the ratio of the largest nonsaturating signal to the standard deviation of the noise under dark conditions is enhanced in WCA scheme. Figure 3a plots the accumulated photoelectrons versus the integration time for three different irradiance signals in normal integration mode. The accumulated photoelectrons increase linearly with the increase in integration time until they reach the full well capacity. The accumulated photoelectrons of a pixel can be expressed as:

(9) $$Q=\{\begin{array}{ll} I\cdot T & \mathrm{if}\;I\leq Q_{MAX}/T\\ Q_{MAX} & \mathrm{otherwise} \end{array}$$

where I is the photocurrent of the incident signal. The largest photocurrent of nonsaturating incident signal is given by, $I_{MAX}=Q_{MAX}/T$.

The total integration time is divided into several time segments when the WCA scheme is utilized. The well capacity is adjusted to a higher value at the beginning of each time segment until it increases to the full well capacity. The accumulated photoelectrons are a piecewise linear function with respect to the integration time. Figure 3b shows that the integration time is divided into two segments, namely, $T_{S}$ and $T-T_{S}$. $T_{S}$ is designated as the adjusting integration time (AIT) at this point. The well capacity is adjusted from $Q_{S}$ to the full well capacity $Q_{MAX}$ at time $T_{S}$. Notably, when the accumulated photoelectrons reach $Q_{S}$ (e.g., the high irradiance signal ($I_{H}$) case in the figure), the output photoelectrons are clipped until time $T_{S}$. The excess photoelectrons will spill over from the lateral overflow gate. However, the accumulated photoelectrons of the low irradiance signal ($I_{L}$) are unaffected. Therefore, the accumulated photoelectrons of a pixel when using the WCA scheme can be expressed as:

(10) $$Q=\{\begin{array}{ll} I\cdot T & \mathrm{if}\;I\leq Q_{S}/T_{S}\\ Q_{S}+I\cdot \left(T-T_{S}\right) & \mathrm{if}\;Q_{S}/T_{S}<I\leq \left(Q_{MAX}-Q_{S}\right)/\left(T-t_{S}\right)T_{S}\\ Q_{MAX} & \mathrm{otherwise} \end{array}$$

The largest photocurrent of incident signal when using the WCA scheme is given by, ${I^{\prime }}_{MAX}=(Q_{MAX}-Q_{S})/(T-T_{S})$. The standard deviation of the noise under dark conditions is the same. Thus, dynamic range is enhanced by a factor:

(11) $$\lambda =\frac{{I^{\prime }}_{MAX}}{I_{MAX}}=\frac{\left(Q_{MAX}-Q_{S}\right)T}{Q_{MAX}\left(T-T_{S}\right)}$$

For a signal that does not saturate after integration, Equation (10) can also be expressed as:

(12) $$Q=IT-\varepsilon \left(I-\frac{Q_{S}}{T_{S}}\right)\cdot \left(IT_{S}-Q_{S}\right)$$

where $\varepsilon \left(t\right)=\{\begin{array}{cc} 1 & t\geq 0\\ 0 & t<0 \end{array}$ is the unit step function. As shown in Equation (12), the exposure parameters $Q_{S}$ and $T_{S}$ explicitly show the effects of output photoelectrons. Therefore, celestial body edge model and star spot imaging model are first established in this study. Then, the influence of exposure parameters on the accuracy of the extraction of optical navigation measurements is analyzed.

### 3.1. Celestial Body Edge Model

**Figure 4:**
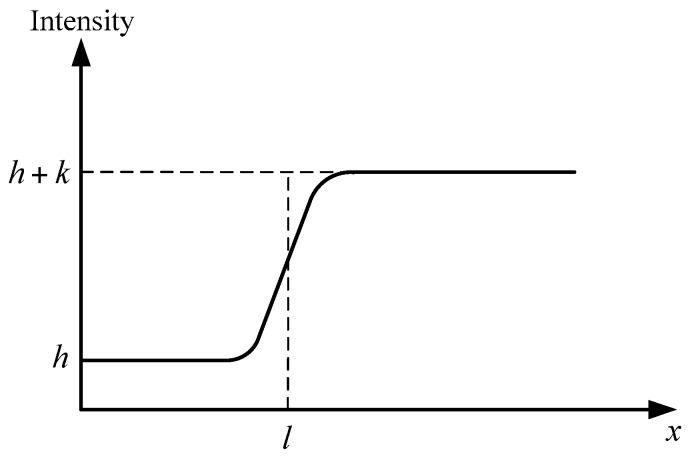
Blurred edge model.

The edge of a celestial body can ideally be modeled as a step function on 1D section. Given the effects of the point spread function (PSF) of the optic system, the real edge adjusts to the blurring effect and is called the blurred edge model. The parameter Gaussian PSF radius $\sigma_{PSF}$ indicates the extent of the blurring effect. The celestial body image is assumed to be an ideal disk, such that the radial energy distribution along the direction normal to the edge is isotropic. Therefore, the 2D imaging model can be regarded as having been formed by 1D edge model that rotates 360° around the center of the disk. A 1D celestial body edge model is established, and the subsequent analyses are based on the said model to simplify the theoretical analysis and calculation. The blurred edge (Figure 4) can be modeled by convolving the ideal step edge with the PSF, which is expressed as [24]:

(13) $$f(x)=\frac{k}{2}\left(erf\left(\frac{x-l}{\sqrt{2}\sigma_{PSF}}\right)+1\right)+h$$

This model has four parameters, namely, background intensity h, edge contrast k, edge location l and Gaussian PSF radius $\sigma_{PSF}$. The background intensity is zero regardless of the random noise. Then, the 1D celestial body edge model is expressed as:

(14) $$f\left(x\right)=\frac{\phi_{P}\eta_{QE}T}{2}\left[erf\left(\frac{x-l}{\sqrt{2}\sigma_{PSF}}\right)+1\right]$$

Thus, the 1D edge gradient model is derived as:

(15) $$f^{\prime }\left(x\right)=\frac{\phi_{P}\eta_{QE}T}{\sqrt{2\pi }\sigma_{PSF}}\exp\left[-\frac{{\left(x-l\right)}^{2}}{2\sigma_{PSF}^{2}}\right]$$

where T is the integration time, $\phi_{P}$ is the incident flux density of the celestial body on the image plane, and $\eta_{QE}$ is the quantum efficiency of the imager sensor. Equation (15) implies that the gradient is maximized at the edge location x=l.

**Figure 5:**
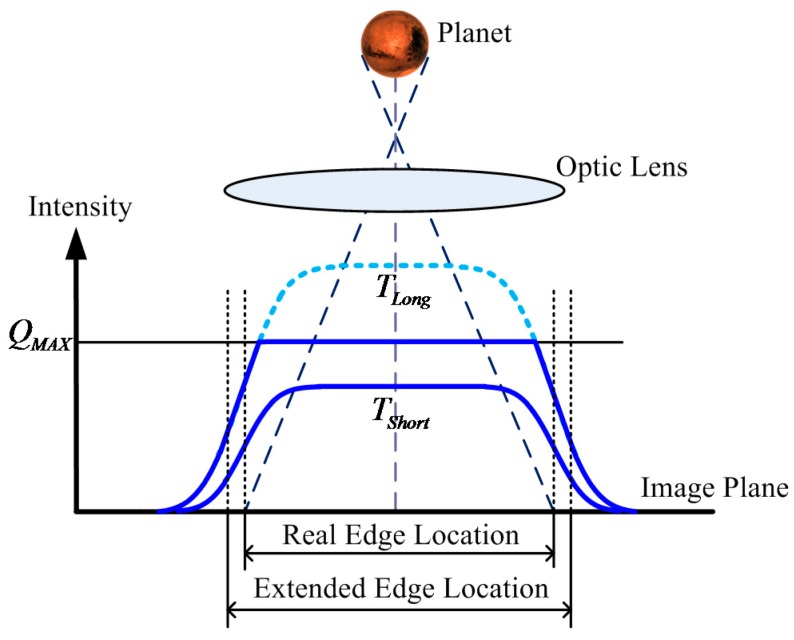
1D profile of the celestial body image in normal integration mode.

In Figure 5, the small amplitude blue solid line denotes the intensity distribution with a short integration time, and the central area is under-saturated. The edge location can be obtained at half the amplitude intersection point. The large amplitude light blue dashed line denotes the intensity distribution where the long integration time should have been. However, the actual intensity distribution is denoted as the blue solid line because the central area is oversaturated. This scenario will result in an extension of the apparent diameter of the celestial body. Therefore, the real edge location cannot be extracted.

**Figure 6:**
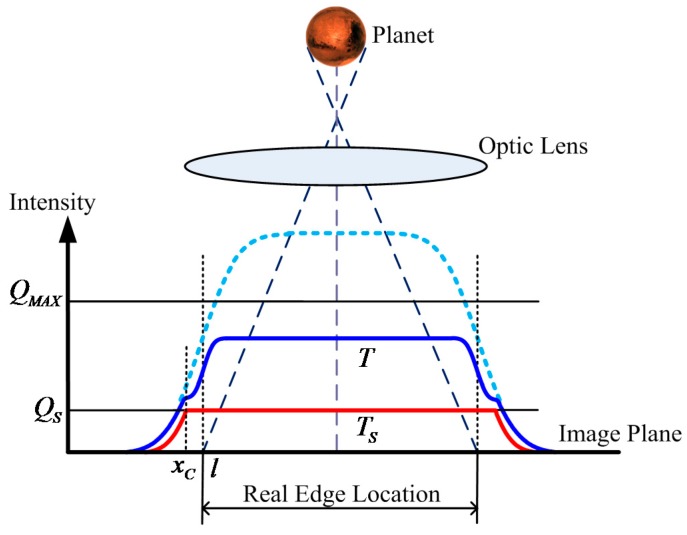
1D profile of the celestial body image when using the WCA scheme.

The WCA scheme is used to avoid oversaturation of the celestial body. Let the total integration time be T and AIT be $T_{S}$. The well capacity is adjusted at time $T_{S}$ from $Q_{S}$ to $Q_{MAX}$. In Figure 6, the red solid line denotes the intensity distribution at time $T_{S}$ and the accumulated photoelectrons in the central area are clipped at $Q_{S}$. The dark blue solid line denotes the intensity distribution at the end of the integration. Compared with Figure 5, the central area of the celestial body image is under-saturated, which avoids the extension of the apparent diameter of the celestial body. However, Figure 6 shows that a shallow energy ring forms around the central area. Therefore, the 1D celestial body edge model becomes a piecewise function when the WCA scheme is applied and can be expressed as:

(16) $$f\left(x\right)=\{\begin{array}{ll} \frac{\phi_{P}\eta_{QE}T}{2}\left[erf\left(\frac{x-l}{\sqrt{2}\sigma_{PSF}}\right)+1\right] & x\leq x_{c}\\ Q_{S}+\frac{\phi_{P}\eta_{QE}\left(T-T_{S}\right)}{2}\left[erf\left(\frac{x-l}{\sqrt{2}\sigma_{PSF}}\right)+1\right] & x>x_{c} \end{array}$$

where $x_{C}$ is the solution of $erf\left(\frac{x-l}{\sqrt{2}\sigma_{PSF}}\right)=\frac{2Q_{S}}{\phi_{P}\eta_{QE}T_{S}}-1$, which denotes the inflection point of the intensity distribution. The irradiance of a celestial body is commonly high, such that $\phi_{P}\eta_{QE}T_{S}$ ≫ $Q_{S}$. Therefore, $x_{C}<l$ is derived.

The 1D edge gradient model when the WCA scheme is utilized can be derived as:

(17) $$f^{\prime }\left(x\right)=\{\begin{array}{cc} \frac{\phi_{P}\eta_{QE}T}{\sqrt{2\pi }\sigma_{PSF}}\exp\left[-\frac{{\left(x-l\right)}^{2}}{2\sigma_{PSF}^{2}}\right] & x\leq x_{C}\\ \frac{\phi_{P}\eta_{QE}(T-T_{S})}{\sqrt{2\pi }\sigma_{PSF}}\exp\left[-\frac{{\left(x-l\right)}^{2}}{2\sigma_{PSF}^{2}}\right] & x>x_{C} \end{array}$$

when x=l, the second term of Equation (17) obtains the maximum value. Therefore, the true edge location can be extracted when the WCA scheme is adopted.

### 3.2. Star Spot Imaging Model

**Figure 7:**
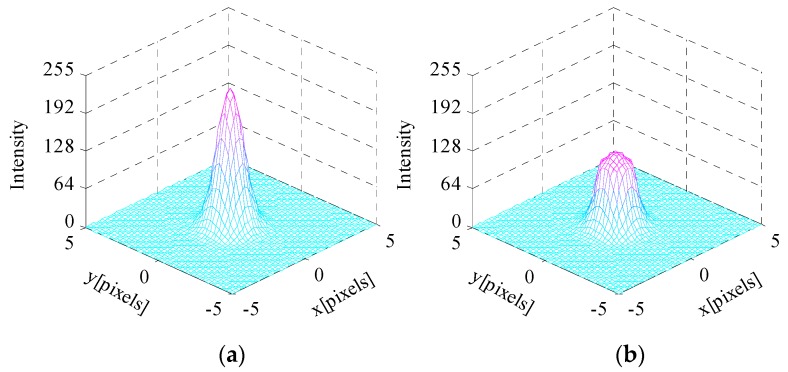
(**a**) Star signal intensity distribution utilizing normal integration mode; and (**b**) star signal intensity distribution using the WCA scheme.

Establishing an accurate star spot imaging model is the first step to achieving high star centroiding accuracy. Stars can be considered point sources at infinity. Stellar rays can be approximated to parallel light rays. Incident stellar lights pass through the optical lens and are focused at a point in the focal plane. However, the lens is generally slightly defocused to improve the centroiding accuracy of a star spot, which spreads to several pixels in the image plane. The profile of a star spot can be described by the Gaussian PSF, whereas the parameter Gaussian PSF radius $\sigma_{PSF}$ indicates the extent of dispersion. When $\sigma_{PSF}$ is high, the region where a star spot spreads out is large. The star spot imaging model in normal integration mode commonly assumes a 2D Gaussian function (Figure 7a) and can be expressed as:

(18) $$E_{nor}(x,y)=\frac{\phi_{S}T\eta_{QE}}{2\pi \sigma_{PSF}^{2}}\exp\left[-\frac{{\left(x-x_{0}\right)}^{2}+{\left(y-y_{0}\right)}^{2}}{2\sigma_{PSF}^{2}}\right]=\Phi_{S}\left(x,y\right)T$$

where $\left(x_{0},y_{0}\right)$ is the true centroid of the star and $\phi_{S}$ is the incident flux of the star on the image plane. $\Phi_{S}\left(x,y\right)=\frac{\phi_{S}\eta_{QE}}{2\pi \sigma_{PSF}^{2}}\exp\left[-\frac{{\left(x-x_{0}\right)}^{2}+{\left(y-y_{0}\right)}^{2}}{2\sigma_{PSF}^{2}}\right]$ is defined as the energy distribution function at this point. The accumulated photoelectrons of a bright star are suppressed when utilizing the WCA scheme, and the excess photoelectrons are drained via the overflow gate. The star spot imaging model when the WCA scheme is applied (Figure 7b) can be expressed as:

(19) $$E_{WCA}(x,y)=\Phi_{S}\left(x,y\right)T-\varepsilon \left(\Phi_{S}\left(x,y\right)-\frac{Q_{S}}{T_{S}}\right)\left(\Phi_{S}\left(x,y\right)T_{S}-Q_{S}\right)$$

The celestial body edge model and star spot imaging model when utilizing the WCA scheme have been established so far. In the subsequent section, the effect of exposure parameters on the accuracy of star centroiding and edge detection is analyzed using the proposed models. Then, the exposure parameters are optimized to obtain the best performance of the navigation sensor utilizing the WCA scheme.

## 4. Celestial Object Image Feature Extraction Accuracy Performance Utilizing the WCA Scheme

The navigation measurements of deep space optical navigation systems are generally combined to calculate the LOS direction and spacecraft location [25]. The accuracy of the apparent diameter and celestial body centroiding is determined by the precision of edge detection. The accuracy of the LOS direction of the navigation sensor is determined by the attitude measurement precision, that is, the centroiding accuracy of the background stars. In this section, the effect of exposure parameters on the accuracy of star centroiding and edge detection is analyzed using the proposed image model, which provides theoretical support for parameter optimization.

### 4.1. Edge Detection Accuracy Performance Utilizing the WCA Scheme

The edge is the part of the image where brightness changes sharply. The edge points based on the blurred edge model are given by the maxima of the first image derivative or zero crossing point of the second image derivative. Steger proposed a subpixel edge extraction algorithm in his doctoral thesis [26]. The basic principle of the algorithm is to perform the second-order Taylor expansion about the pixel where the local gradient is maximized in the direction of the edge normal and to determine the subpixel location of the zero crossing point of the second derivative. In this study, the edge of the celestial body is extracted using this algorithm, which is essentially a fitting interpolation algorithm.

Given that the edge detection algorithm is based on image derivative information, it is highly sensitive to noise. Therefore, the image derivatives must be estimated by convolving the image with the derivatives of the Gaussian smoothing kernel. Edges appear as bright lines in an image that contains the absolute value of the gradient. The second-order Taylor expansion about the maximized gradient pixel in the direction of the edge normal is expressed as:

(20) $$r^{\prime }\left(x\right)=r^{\prime }\left(x_{0}\right)+r^{\prime \prime }\left(x_{0}\right)x+\frac{1}{2}r^{\prime \prime \prime }\left(x_{0}\right)x^{2}$$

where $x_{0}$ is the pixel center. The subpixel location of the edge where $r^{\prime \prime }\left(x\right)=0$ is expressed as:

(21) $$l_{0}=x_{0}-\frac{r^{\prime \prime }\left(x_{0}\right)}{r^{\prime \prime \prime }\left(x_{0}\right)}$$

The 1D celestial body edge model is a piecewise function when the WCA scheme is applied. The gradient of the edge model is derived by convolving the edge model with the first derivative of the Gaussian smoothing kernel and expressed as:

(22) $$\begin{array}{l} r^{\prime }\left(x\right)=f\left(x\right)*g^{\prime }\left(x\right)=\int_{-\infty }^{\infty }f^{\prime }\left(\tau \right)g\left(x-\tau \right)d\tau \\ =\int_{-\infty }^{x_{c}}\frac{\phi_{P}\eta_{QE}T}{\sqrt{2\pi }\sigma_{PSF}}\exp\left[-{\left(\frac{\tau -l}{\sqrt{2}\sigma_{PSF}}\right)}^{2}\right]\cdot \frac{1}{\sqrt{2\pi }\sigma }\exp\left[-{\left(\frac{x-\tau }{\sqrt{2}\sigma }\right)}^{2}\right]d\tau +\\ \int_{x_{c}}^{\infty }\frac{\phi_{P}\eta_{QE}\left(T-T_{S}\right)}{\sqrt{2\pi }\sigma_{PSF}}\exp\left[-{\left(\frac{\tau -l}{\sqrt{2}\sigma_{PSF}}\right)}^{2}\right]\cdot \frac{1}{\sqrt{2\pi }\sigma }\exp\left[-{\left(\frac{x-\tau }{\sqrt{2}\sigma }\right)}^{2}\right]d\tau \\ =\frac{\phi_{P}\eta_{QE}}{\sqrt{2\pi (\sigma_{PSF}^{2}+\sigma^{2})}}\exp\left[-\frac{{\left(x-l\right)}^{2}}{2\left(\sigma_{PSF}^{2}+\sigma^{2}\right)}\right]\left\{T-\frac{T_{S}}{2}+\frac{T_{S}}{2}erf\left[\Theta (x)\right]\right\} \end{array}$$

Thus, the second derivative of edge model is derived as:

(23) $$\begin{array}{l} r^{\prime \prime }(x)=-\frac{\phi_{P}\eta_{QE}(x-l)}{\sqrt{2\pi (\sigma_{PSF}^{2}+\sigma^{2})}(\sigma_{PSF}^{2}+\sigma^{2})}\exp\left[-\frac{{\left(x-l\right)}^{2}}{2\left(\sigma_{PSF}^{2}+\sigma^{2}\right)}\right]\left\{T-\frac{T_{S}}{2}+\frac{T_{S}}{2}erf\left[\Theta (x)\right]\right\}\\ -\frac{\phi_{P}\eta_{QE}T_{S}\sigma_{PSF}}{2\pi \sigma (\sigma_{PSF}^{2}+\sigma^{2})}\exp\left[-\frac{{\left(x-l\right)}^{2}}{2\left(\sigma_{PSF}^{2}+\sigma^{2}\right)}\right]\exp\left\{-\Theta^{2}(x)\right\} \end{array}$$

where $\Theta (x)=\frac{\sigma_{PSF}^{2}(x_{c}-x)+\sigma^{2}(x_{c}-l)}{\sqrt{2(\sigma_{PSF}^{2}+\sigma^{2})}\sigma_{PSF}\sigma }$. The edge location is the zero crossing point of the second derivative, which indicates that the solution of Equation (23) and $r^{\prime \prime \prime }\left(x\right)r^{\prime }\left(x\right)<0$ are required. Given that the analytical solution cannot be calculated, the effect of exposure parameters on the edge detection accuracy is analyzed by numerical simulations. The edge localization error is the function of the Gaussian radius $\sigma_{PSF}$, well capacity $Q_{S}$, total integration time T, AIT $T_{S}$, Gaussian smoothing kernel radius σ, and edge location l. For a given optical system, $\sigma_{PSF}$ is constant, σ is a parameter of the edge detection algorithm, and T is determined by the limiting detectable star visual magnitude for the navigation sensor. This study focuses on analyzing the edge detection error caused by $Q_{S}$ and $T_{S}$. Systematic error in edge detection is also introduced by pixelization. However, l is a random variable in practice that is uniformly distributed over a pixel. The root mean square error is defined as the error of edge detection, which is expressed as:

(24) $$\delta_{E}(Q_{S},T_{S})={\left[\int_{-0.5}^{0.5}\delta^{2}(Q_{S},T_{S},l)dl\right]}^{2}$$

**Figure 8:**
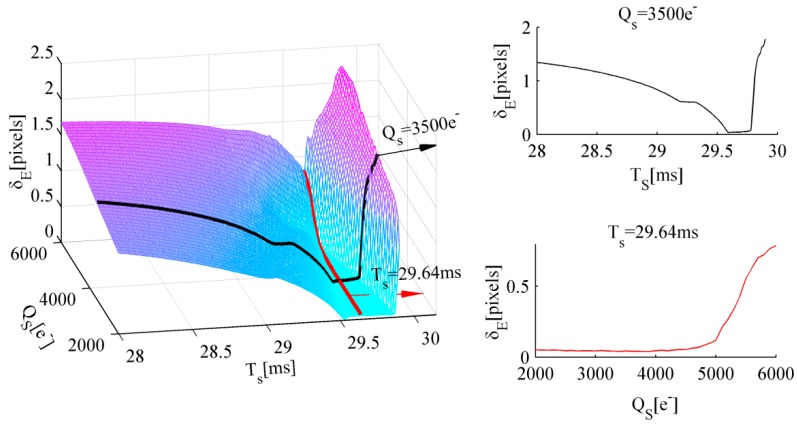
Edge detection error $\delta_{E}$ (**left**) versus well capacity $Q_{S}$ and AIT $T_{S}$ (**right**).

$\sigma_{PSF}=0.67$ pixels, σ = 0.55 pixels, T=30 ms are set at this point. Then, the simulated celestial body images with temporal noise and fixed pattern noise are generated. Sources of noises which are taken into consideration include photon shot noise, dark current noise, readout noise, quantization noise, dark signal non-uniformity and photon response non-uniformity. The full well capacity is set to $Q_{MAX}=15,000e^{-}$ consist with the image sensor we utilized. The edge detection error simulation results are shown in Figure 8.

First, the relationship between edge detection error $\delta_{E}$ and AIT $T_{S}$ is discussed. The black line indicates that $\delta_{E}$ is evidently affected by $T_{S}$. An interval exists wherein $\delta_{E}$ is significantly small. The second segment of the integration time $T-T_{S}$ is relatively long when $T_{S}$ is short, which leads to the oversaturation of the central region pixels and extension of the apparent diameter of the celestial body. However, $T\;-\;T_{S}$ is relatively short when $T_{S}$ is excessively long, which leads to a small intensity contrast between the central region and the energy ring. The algorithm will extract the edge of the “rings” instead of the actual edge location. Therefore, $\delta_{E}$ initially reaches the minimum number and then increases with the increase in $T_{S}$.

**Figure 9:**
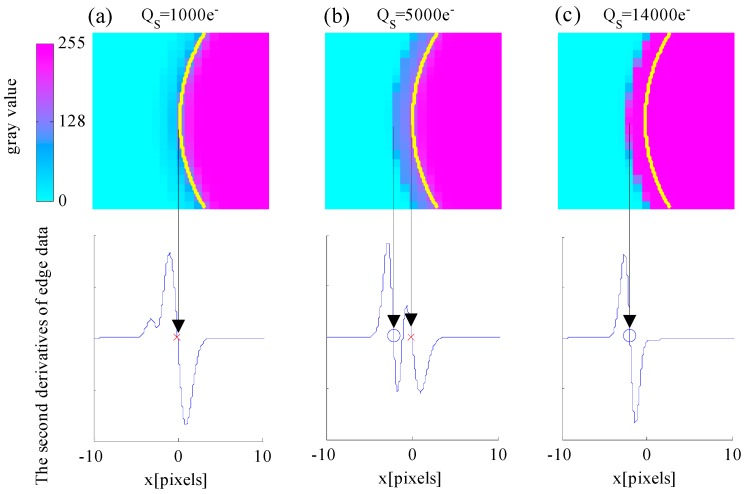
Effect of different well capacity values on the edge detection results when: (**a**) $Q_{S}=1000e^{-}$; (**b**) $Q_{S}=5000e^{-}$; and (**c**) $Q_{S}=14,000e^{-}$.

Second, the relationship between edge detection error $\delta_{E}$ and well capacity $Q_{S}$ is discussed. Setting the AIT to an appropriate value leads to a relatively small $\delta_{E}$. $T_{S}=29.6\;\mathrm{ms}$ is set at this point. The red line indicates that $\delta_{E}$ varies slightly and remains nearly constant at the beginning with the increase in $Q_{S}$. As $Q_{S}$ continues to increase, $\delta_{E}$ increases sharply. The reason for this relationship is provided in Figure 9, which shows the simulated images of the celestial object and the second derivative of the edge model expressed in Equation (23) when the well capacity values are 1000$e^{-}$, 5000$e^{-}$and 14,000$e^{-}$.

In Figure 9, the purple color represents the saturated pixels, whereas the cyan color represents the pixels with zero intensity. The yellow arc represents the true edge of the celestial body. The red cross symbol indicates the zero-crossing point of the second derivative, which is located at the true edge location, whereas the blue circle symbol indicates the zero-crossing point that deviates from the true edge location. Figure 9a shows that the intensity of the energy ring is small when the well capacity $Q_{S}$ is small and that the zero-crossing points can be extracted at the actual edge location. Figure 9b shows that the intensity of the energy ring increases with the increase in $Q_{S}$ and that the zero crossing points exist at the location of the energy ring. The algorithm extracts double edges, and the false edge can be rejected. Figure 9c shows that the intensity of the energy ring is higher with a larger $Q_{S}$ and that no zero-crossing points are obtained at the actual edge location. The extracted edge deviates from the true location. Thus, a large $Q_{S}$ value results in false edge extraction.

Thus, the well capacity and AIT are the main factors that affect the edge detection error. $T_{S}$ evidently influences on the accuracy of edge detection. The edge detection error initially reaches the minimum number and then increases with the increase in $T_{S}$; $Q_{S}$ must not be excessively large.

### 4.2. Star Centroiding Accuracy Performance Utilizing the WCA Scheme

Star centroiding accuracy is the basis for attitude accuracy. The star centroiding accuracy performance is analyzed when the WCA scheme is employed to ensure attitude accuracy. The total centroiding error is decomposed into the x-and y-component errors. The errors in each case can be proven to be the same. Thus, this study focuses on analyzing the x-component errors as an example.

The centroiding error of the x-component $\delta_{x}$ when the WCA scheme is applied is expressed as:

(25) $$\begin{array}{l} \delta_{x}=\frac{\sum_{i}\sum_{j}x_{i}I_{ij}}{\sum_{i}\sum_{j}I_{ij}}-x_{0}\\ \;=\frac{\sum_{i}\sum_{j}\Phi_{S}\left(x_{i},y_{j}\right)Tx_{i}-\sum_{i}\sum_{j}x_{i}\cdot \varepsilon \left(\Phi_{S}\left(x_{i},y_{j}\right)-\frac{Q_{S}}{T_{S}}\right)\cdot \left[\Phi_{S}\left(x_{i},y_{j}\right)T_{S}-Q_{S}\right]}{\phi_{S}\eta_{QE}T-\sum_{i}\sum_{j}\varepsilon \left(\Phi_{S}\left(x_{i},y_{j}\right)-\frac{Q_{S}}{T_{S}}\right)\cdot \left[\Phi_{S}\left(x_{i},y_{j}\right)T_{S}-Q_{S}\right]}-x_{0} \end{array}$$

In Equation (25), $\delta_{x}$ is the function of the Gaussian radius $\sigma_{PSF}$, well capacity $Q_{S}$, total integration time T, AIT $T_{S}$, incident flux of the star on the image plane $\phi_{S}$, and actual star location $x_{0}$. T is determined by the limiting detectable star visual magnitude for the navigation sensor. If $x_{0}$ moves within a pixel, then $\delta_{x}$ changes periodically. However, $x_{0}$ is a random variable that is uniformly distributed over a pixel within the range [−0.5, 0.5) in practice. The root mean square error is defined as the error of x, as:

(26) $$\delta_{x,S}(Q_{S},T_{S})={\left[\int_{-0.5}^{0.5}\delta_{x}^{2}(Q_{S},T_{S},x_{0})dx_{0}\right]}^{2}$$

**Figure 10:**
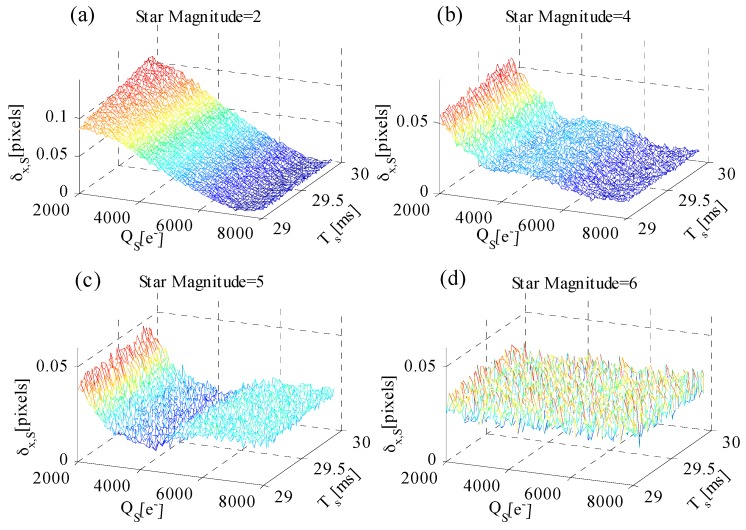
$\delta_{x,S}$ versus well capacity $Q_{S}$ and AIT $T_{S}$ for different star magnitudes: (**a**) star magnitude = 2; (**b**) star magnitude = 4; (**c**) star magnitude = 5; and (**d**) star magnitude = 6.

After adding temporal noise and fixed pattern noise to the simulated star image, the relationship among $\delta_{x,S}$, $Q_{S}$, and $T_{S}$ is analyzed with different star magnitudes. Figure 10 shows the simulated results when the star magnitude is 2, 4, 5, and 6. First, the relationship between star centroiding error $\delta_{x,S}$ and AIT $T_{S}$ is discussed. Figure 10a shows that the centroiding error of the star magnitude = 2 slowly increases with the increase in $T_{S}$. Figure 10b–d shows that the centroiding error variation caused by $T_{S}$ can be neglected. Thus, $\delta_{x,S}$ is less affected by $T_{S}$. Second, the relationship between star centroiding error $\delta_{x,S}$ and well capacity $Q_{S}$ is discussed. Figure 10a–c shows that $\delta_{x,S}$ decreases with the increase in $Q_{S}$. However, the centroiding error variation of a dim star (Figure 10d) caused by well capacity can be neglected.

The total star centroiding error is expressed as:

(27) $$\delta_{S,cen}=\sqrt{\delta_{x,S}^{2}+\delta_{y,S}^{2}}$$

The relationship between total star centroiding error and exposure parameters is consistent with the x-component errors. Thus, the centroiding error of a dim star is unaffected by the WAC scheme. The centroiding error of a bright star decreases with the increase in well capacity, and the AIT effect can be ignored.

**Figure 11:**
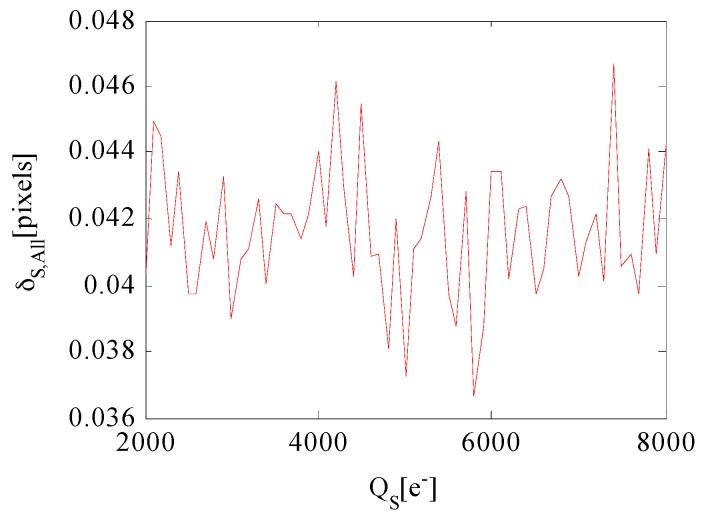
Overall star centroiding error versus well capacity.

In the preceding sections, the centroiding accuracy performance of a single star is analyzed when the WCA scheme is adopted. Many stars in the FOV are required to increase the attitude determination accuracy in practice. More dim stars have been generally recorded than bright stars in the FOV. The overall star centroiding error is defined as the weighted average of centroiding errors for different star magnitudes at this point. The overall centroiding error can directly reflect the attitude determination accuracy of the optical navigation sensor. The star magnitudes range from 0 to 7 at 0.5 intervals. The star magnitudes that range from $m_{V}\;-\;0.25$ to $m_{V}\;+\;0.25$ are considered the magnitude $m_{V}$ to simplify the analysis process. Therefore, the overall star centroiding error is expressed as:

(28) $$\delta_{S,All}=\frac{\sum_{i=0}^{7}\delta_{S,M_{Vi}}(N_{M_{Vi},FOV}-N_{M_{Vi-1},FOV})}{N_{M_{V7},FOV}}$$

where $N_{M_{Vi},FOV}$ is the average number of stars brighter than magnitude $M_{Vi}$ in the FOV, and is expressed as [27]:

(29) $$N_{M_{Vi},FOV}=6.57\cdot e^{1.08M_{Vi}}\cdot \frac{1-\cos\left(A/2\right)}{2}$$

where A is the FOV size. The number of stars in the FOV increases exponentially with the increase in star magnitude $M_{Vi}$. There are much more dim stars than the bright ones in the FOV. As a result, the star centroiding error of dim star contributes more to the overall centroiding error. Figure 11 shows the relationship between overall centroiding error and well capacity $Q_{S}$ when $T_{S}\;=\;29.6\;\mathrm{ms}$. The overall centroiding error fluctuation is relatively stable with the increase in $Q_{S}$ because variation of star centroiding error of dim star caused by $Q_{S}$ is less affected than bright star Therefore, the variation of overall centroiding error caused by well capacity can be neglected.

In summary, the well capacity $Q_{S}$ is mainly responsible for the centroiding error of a single star. However, the attitude accuracy affected by well capacity can be neglected.

## 5. Exposure Parameter Optimization

In the preceding sections, the celestial body edge model and star spot image model are established. Both models are piecewise functions, such that the analytical solution of the optimal exposure parameter is difficult to obtain. Thus, the optimal exposure parameters are obtained by conducting Monte Carlo simulation. The exposure parameters include the total integration time T, AIT $T_{S}$, and well capacity $Q_{S}$. An appropriate value of T ensures that sufficient stars are covered in the FOV for star pattern recognition, which is a prerequisite for a reliable attitude measurement function. The total integration time is generally set to a relatively longer value to ensure that dim stars can be identified as reliable, which can cause the target celestial body to become overexposed. Therefore, the WCA scheme is adopted, and the AIT and well capacity are optimized to obtain the best navigation performance. The optical system employed in this study has the following parameters: aperture D = 40 mm, focal length f = 100 mm, and optical transmission τ = 80%. The image sensor utilized is CMV20000, the parameters of which are listed in Table 1.

### 5.1. Total Integration Time T

The total integration time is determined by the limiting detectable star visual magnitude for the navigation sensor. Navigation sensors must conduct star pattern recognition to obtain attitude information. A sufficient number of navigation stars must be present in the FOV to ensure the effectiveness of the star pattern recognition algorithm. A star can be generally identified as reliable if the SNR of at least five pixels are more than 5. Thus, T must satisfy that the SNR of the darkest pixel of limiting detectable star is more than 5, which can be expressed as:

(30) $$SNR=\frac{K\phi_{S}\eta_{QE}T}{N_{noise}}=\frac{K\phi_{S0}\eta_{QE}T\cdot 2.512^{-M_{V}}}{\sqrt{n_{Shot}^{2}+n_{Dark}^{2}+n_{PRNU}^{2}+n_{DSNU}^{2}+n_{read}^{2}+n_{ADC}^{2}}}>5$$

Thus, the following expression can be derived:

(31) $$T>\frac{5\cdot \sqrt{n_{Shot}^{2}+n_{Dark}^{2}+n_{PRNU}^{2}+n_{DSNU}^{2}+n_{read}^{2}+n_{ADC}^{2}}}{K\phi_{S0}\eta_{QE}\cdot 2.512^{-M_{V}}}$$

where $n_{Shot}$, $n_{Dark}$, $n_{PRNU}$, $n_{DSNU}$, $n_{read}$, and $n_{ADC}$ denote the standard deviations of photon shot noise, dark current noise, photon response non-uniformity noise (PRNU), dark signal non-uniformity noise (DSNU), readout noise and quantization noise, respectively. Photon shot noise follows a Poisson distribution and is dependent on incident flux on the pixel; its variance is equal to the counts of photoelectrons in the imaging process. Dark current noise also follows a Poisson distribution, and its variance is equal to the production of dark current and exposure time. The standard deviation of the quantization noise is equal to $n_{ADC}=1/\sqrt{12}G$, where G denote the conversion gain. These noise terms can be derived from the parameters in Table 1. K is the ratio of the energy of the darkest pixel to the total energy of the star signal. K=0.0287 is set at this point. $\phi_{S0}=5.4\;\times \;10^{4}\mathrm{photons}/\mathrm{ms}$ is the incident flux of the star, whose magnitude is 0. $M_{V}$ is the limiting detectable star visual magnitude for the navigation sensor. $M_{V}=6$ is set at this point. Equation (31) shows that the total integration time must satisfy T>25.4 ms. T=30 ms is set to provide the system with a certain degree of redundancy.

### 5.2. Adjusting Integration Time $T_{S}$

The AIT $T_{S}$ is one of the main factors that influences the edge detection accuracy performance of the celestial body. The edge detection error initially reaches the minimum number and then increases with the increase in $T_{S}$ based on the previous conclusion. However, the variation of star centroiding error caused by $T_{S}$ can be neglected. Therefore, this study focuses on optimizing $T_{S}$ to minimize the edge detection error.

Monte Carlo simulation is performed through the following procedure: First, a total of 8000 groups of celestial body images are generated using the proposed model. The well capacity values range from 3000$e^{-}$ to 5000$e^{-}$ at 25$e^{-}$ intervals. The AIT values range from 29.6 ms to 29.8 ms at 0.002 ms intervals. Each group contains 100 celestial body images, whose individual center coordinates are fixed; then, random noise is added. However, the radius of the celestial body obeys uniform distribution over a pixel, which is equivalent to the edge location that changes uniformly over a pixel. Second, edge data of the 100 celestial bodies are extracted using the edge detection algorithm. The absolute fitting radius error is obtained by fitting these edge points utilizing the least square circle fitting algorithm. The absolute radius error is a direct expression of the edge detection error. Then, the standard deviation of these absolute radius errors in one group is considered the average edge detection error at certain well capacity and AIT values. Finally, this procedure is repeated until all groups of celestial body images are processed.

**Figure 12:**
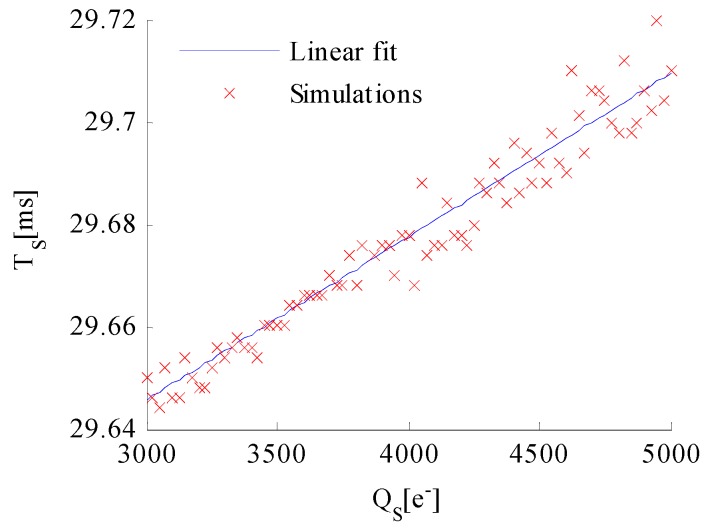
Simulation results of the optimal $T_{S}$.

The simulation conditions are set as follows: the incident flux of the celestial body on the image plane average in one pixel $\phi_{P}\;=\;7.5\;\times \;10^{4}\;\mathrm{photons}/\mathrm{ms}$, the total integration time T=30 ms, the Gaussian radius $\sigma_{PSF}\;=\;0.67$ pixels, and the Gaussian smoothing kernel radius σ = 0.55 pixels. The AIT $T_{S}$ that corresponds to the minimum standard deviation of the edge detection error is selected as the optimal $T_{S}$ at a certain well capacity, which is shown as a red scattered point in Figure 12.

The simulation results are shown in Figure 12. The blue solid line indicates the linear function between well capacity $Q_{S}$ and optimal $T_{S}$ fitted by MATLAB. The optimal $T_{S}$ increases with the increase in well capacity. Therefore, the optimal AIT is derived as:

(32) $$T_{S}=T-\frac{Q_{MAX}-Q_{S}}{\phi_{P}\eta_{QE}}$$

The same conclusion can also be obtained under other simulation conditions. Thus, the optimal AIT $T_{S}$ when utilizing the WCA scheme is given by Equation (32).

### 5.3. Well Capacity $Q_{S}$

The optimization of well capacity $Q_{S}$ is determined by two conditions. First, if $Q_{S}$ is set excessively low, then the SNR of a dim star decreases, which can cause the star identification failure of the limiting detectable star. Therefore, providing the lower limit of the well capacity is necessary. Second, $Q_{S}$ is the main factor that affects the accuracy of edge detection when the WCA scheme is applied, and the optimal $Q_{S}$ must minimize the edge detection error. Therefore, the optimal well capacity $Q_{S}$ is comprehensively determined by the aforementioned conditions.

The intensity distribution of the limiting detectable star cannot be degraded to ensure that it is identified reliably. Thus, the lower limit of well capacity is expressed as:

(33) $$Q_{S}\geq T_{S}K_{B}\phi_{S0}\eta_{QE}\cdot 2.512^{-M_{Vt}}$$

By substituting Equation (32) into Equation (33), the following expression is obtained:

(34) $$Q_{S}\geq \frac{\left(\phi_{P}T\eta_{QE}-Q_{MAX}\right)K_{B}\phi_{S0}\cdot 2.512^{-M_{Vt}}}{\phi_{P}-K_{B}\phi_{S0}\cdot 2.512^{-M_{Vt}}}$$

where $K_{B}$ is the ratio of the energy of the brightest pixel to the total energy of the star signal, $K_{B}=0.2965$ is set at this point. $M_{Vt}$ is the star magnitude limit threshold, and the SNR of a star that is dimmer than magnitude $M_{Vt}$ does not decrease when the WCA scheme is employed. $M_{Vt}\;=\;5.5$ is set at this point, and $Q_{S}\geq \;1400e^{-}$ is obtained.

The well capacity $Q_{S}$ is another factor that influences the edge detection performance of the celestial body. Monte Carlo simulation is performed to obtain the optimal well capacity value. Given that the relationship between optimal AIT and well capacity is already derived in the previous section, the optimal exposure parameters under current imaging conditions are determined. A total of 300 groups of celestial body images were generated based on the celestial body edge model. Then, random noise is added. The well capacity values range from 2000$e^{-}$ to 5000$e^{-}$ at 10$e^{-}$ intervals. Each $T_{S}$ is derived using Equation (32) at a certain $Q_{S}$. Then, the edge data of the 100 celestial bodies are extracted using the edge detection algorithm. The absolute fitting radius error is obtained by fitting these edge points utilizing the least square circle fitting algorithm. The standard deviation of these absolute radius errors in one group is considered the average edge detection error at a certain well capacity. The well capacity $Q_{S}$ that corresponds to the minimum standard deviation of the edge detection error is selected as the optimal $Q_{S}$ if it also satisfies the condition expressed in Equation (34).

**Figure 13:**
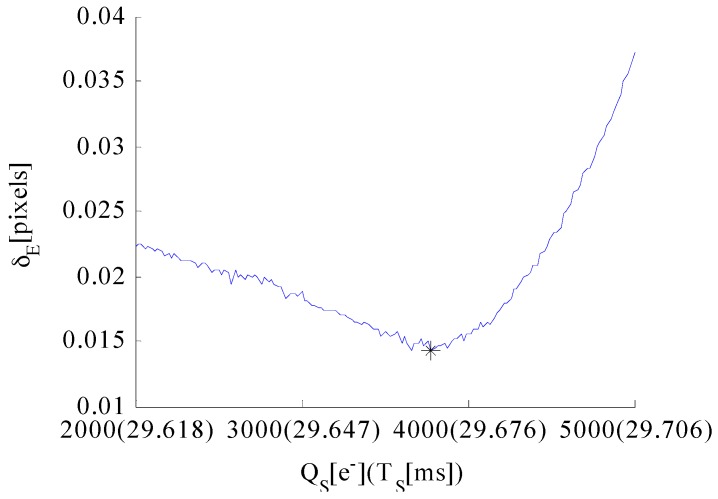
Simulation results of the optimal $Q_{S}$.

The simulation results of the optical system in this study are shown in Figure 13. The abscissa denotes the well capacity $Q_{S}$, whereas the values in the parentheses denote the optimal $T_{S}$ that correspond to the current $Q_{S}$. The edge detection error initially reaches the minimum number and then increases with the increase in $Q_{S}$. The symbol “*” indicates the optimal $Q_{S}$. Thus, the optimal solutions for the exposure parameters are identified. The optimal well capacity of the navigation sensor of the optical system employed in this study is 3750$e^{-}$, whereas the optimal AIT is 29.67 ms.

## 6. Experimental Results and Analysis

Laboratorial single-star imaging and accuracy analysis experiment and night sky experiment are conducted to validate the correctness of the proposed models, accuracy performance analysis, and optimal exposure parameters. The image sensor of the navigation sensor adopted in these experiments is CMV20000, the parameters of which are listed in Table 1.

### 6.1. Laboratorial Single-Star Imaging and Accuracy Analysis Experiment

**Figure 14:**
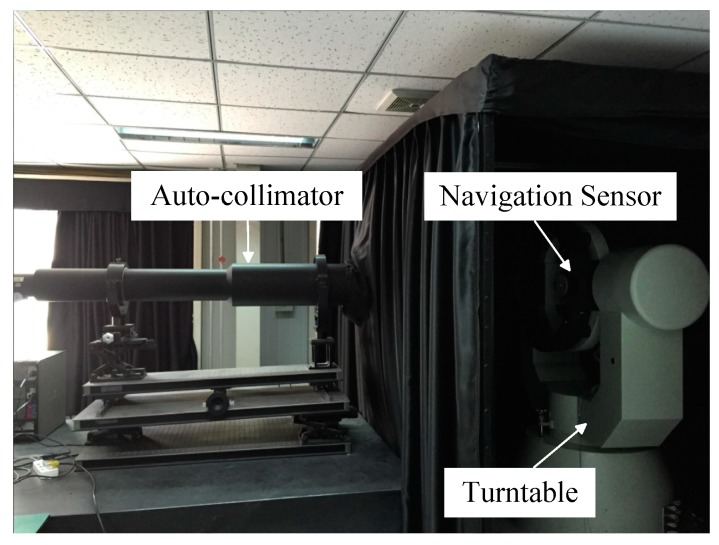
Setup for the laboratorial experiment.

A laboratorial single-star imaging and accuracy analysis experiment is performed to validate the star spot imaging model and centroiding accuracy performance when the WCA scheme is employed. The autocollimator in the laboratory is used to generate infinite distance star signals with different star magnitudes. The navigation sensor is mounted on a turntable, as shown in Figure 14.

**Figure 15:**
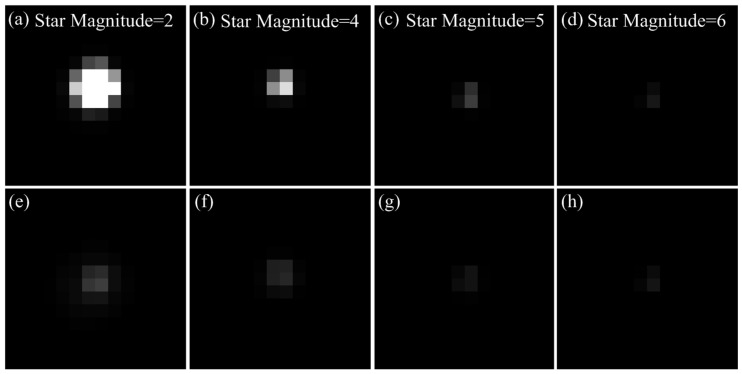
(**a**–**d**) Star images of magnitudes 2, 4, 5 and 6 when using the normal integration mode; and (**e**–**h**) star images of magnitudes 2, 4, 5 and 6 when using the WCA scheme.

The exposure parameters of the navigation sensor are set as follows: total integration time T=30 ms, AIT $T_{S}=29.67\;\mathrm{ms}$, and well capacity $Q_{S}=3750e^{-}$. Figure 15 shows the star images of different magnitudes using the normal integration mode and the WCA scheme. The brightness degree of the bright star clearly degraded when the WCA scheme is applied. However, the energy distribution of the dim star is unaffected. Therefore, the star point imaging model is validated by the experiment.

**Figure 16:**
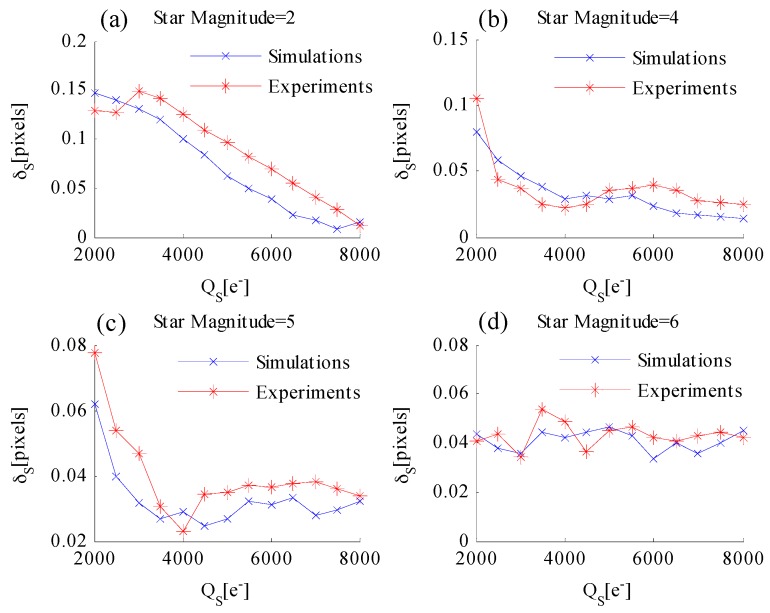
Star centroiding error versus well capacity: (**a**) star magnitude = 2; (**b**) star magnitude = 4; (**c**) star magnitude = 5; and (**d**) star magnitude = 6.

The centroiding accuracy performance when utilizing the WCA scheme is validated. The exposure parameters of the navigation sensor are set as follows: total integration time T=30 ms, AIT $T_{S}=29.67\;\mathrm{ms}$, and well capacity $Q_{S}$ ranges from 2000$e^{-}$ to 8000$e^{-}$ at 500$e^{-}$ intervals. Then, star centroiding is performed and experimental data are recorded. Although the true position of the star is unknown, the average centroid position $\left({\bar{x}}_{cen},{\bar{y}}_{cen}\right)$ of a bright unsaturated star image can be considered the estimated value of the true position. Then, the standard deviations of the centroiding error of each magnitude with respect to position $\left({\bar{x}}_{cen},{\bar{y}}_{cen}\right)$ are calculated at each certain $Q_{S}$. The experimental results are shown in Figure 16.

In Figure 16, the experimental results are denoted with red solid lines, whereas the simulation results are denoted with blue solid lines. The centroiding accuracy performance from the experiment is consistent with the simulation results. Thus, the laboratorial single-star imaging and accuracy analysis experiment validates the conclusions of this study.

### 6.2. Night Sky Observation and Accuracy Analysis Experiment

**Figure 17:**
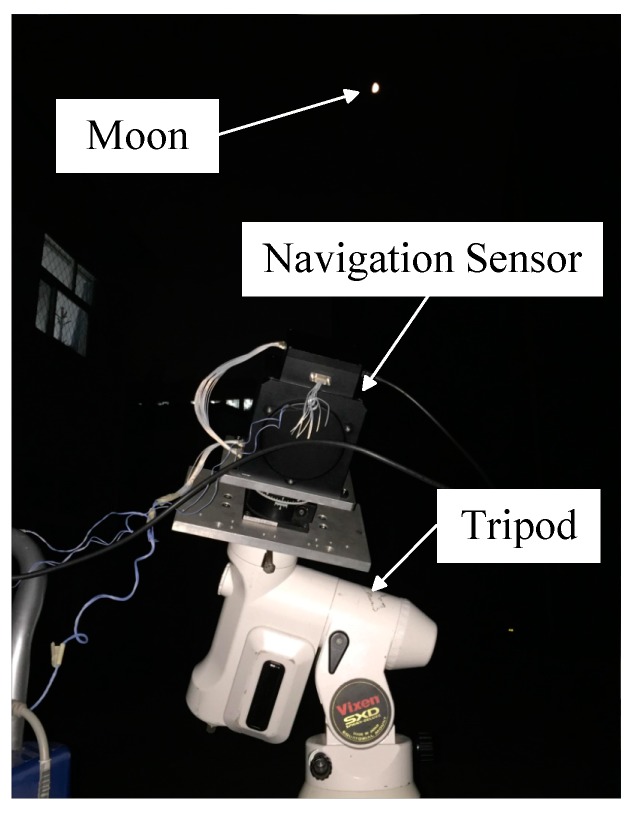
Setup of the night sky experiment.

A night sky observation and accuracy analysis experiment is performed to validate the correctness of the celestial body edge model and optimal exposure parameters when the WCA scheme is applied. Moreover, whether the optical navigation measurements of the stars and target celestial body from the image by a single exposure with the optimal exposure parameters satisfy the requirements of navigation accuracy is validated. In Figure 17, the navigation sensor is installed on a tripod. The hardware configurations of the navigation sensor are the same as those previously mentioned.

#### 6.2.1. Observations of the Moon and Accuracy Analysis Experiment

**Figure 18:**
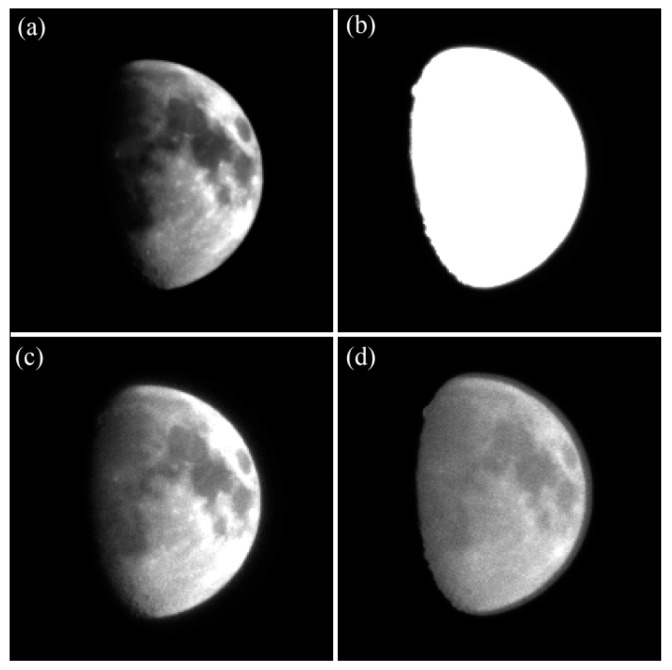
Lunar images with different exposure parameters: (**a**) T=0.4 ms utilizing the normal integration mode; (**b**) T=30 ms utilizing the normal integration mode; (**c**) T=30 ms, $T_{S}=29.67\;\mathrm{ms},$ $Q_{S}=3750e^{-}$ utilizing the WCA scheme; and (**d**) T=30 ms, $T_{S}=29.67\;\mathrm{ms},$ $Q_{S}=6093e^{-}$ utilizing the WCA scheme.

The images of the Moon are obtained utilizing the normal integration and WCA schemes, as shown in Figure 18. Figure 18b shows that the image is largely overexposed, and the apparent diameter of the Moon is extended significantly. Figure 18c shows the Moon imaged with optimal exposure parameters utilizing the WCA scheme. Compared with the image shown in Figure 18b, although the total integration time is the same, the image of the Moon is well exposed, and the image exhibited suitable performance of navigation measurement extraction when the WCA scheme is applied. Figure 18d shows the image of the Moon when the well capacity is set to $6093e^{-}$. The energy ring around the Moon is clearly visible. The observation results validate the correctness of the celestial body edge model when the WCA scheme is adopted.

**Figure 19:**
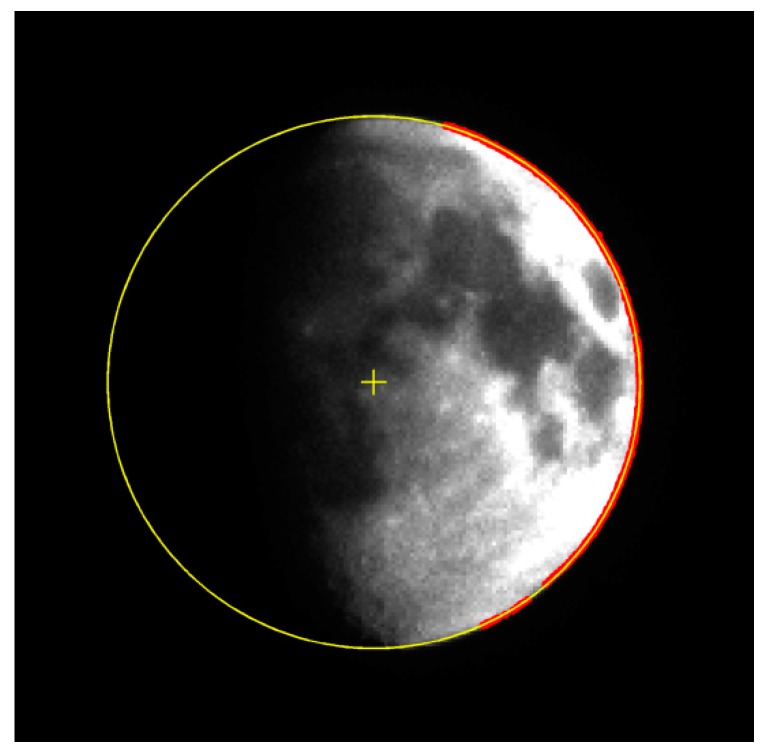
Edge detection and circle fitting results of the Moon image.

The theoretical value of the apparent diameter of the Moon is estimated to be 71.02 pixels on the image plane by applying the STK software to simulate the distance from the observation location to the Moon. The edge detection algorithm is employed to extract the edge of the Moon. The least squares circle fitting is utilized to obtain the apparent radius and centroid of the Moon image. The results are shown in Figure 19.

In Figure 19, the red scatter points are the extracted edge points, the yellow circle is the fitting circle, and “+” symbol is the centroid position. The minimum relative deviation of the apparent radius with respect to the theoretical value is obtained when the optimal exposure parameters are adopted. Table 2 shows the average extraction results of the apparent radius under different exposure conditions. However, the apparent radius errors are larger than the simulation results. This phenomenon may be attributed to atmospheric turbulence and lens calibration error, which are beyond the scope of this study. Thus, these factors are not considered in the model. In summary, the Moon observations validate the reliability of error analysis and parameter optimization.

#### 6.2.2. Observations of the Moon and Stars in the Same FOV

**Figure 20:**
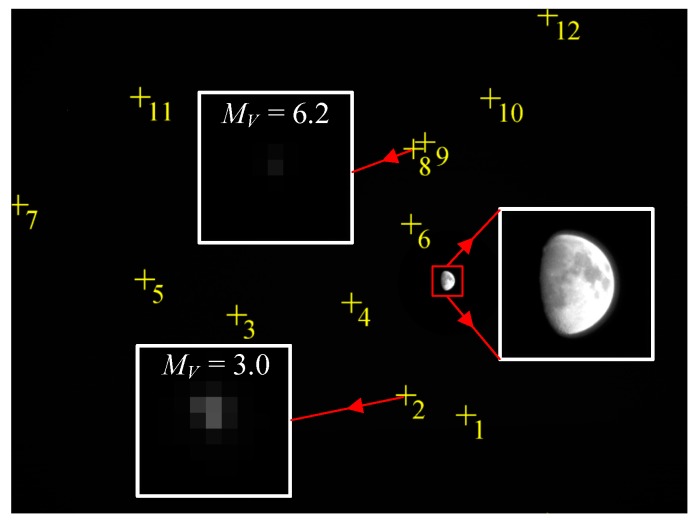
Observations of the Moon and stars in the same FOV.

Images of stars and the Moon in the same FOV are taken utilizing the WCA scheme and optimal exposure parameters, as shown in Figure 20. The star pattern identification algorithm is applied, and the identified stars are denoted with the yellow “+” symbol. A total of 12 stars are identified. The brightest star magnitude is 3.0, whereas the dimmest star magnitude is 6.2. The centroid positions and magnitude of the identified stars in the image are listed in Table 3.

Under the optimal exposure parameters, the navigation sensor can simultaneously identify the stars that are dimmer than the limiting detectable star magnitude and extract the high-accuracy edge location of the celestial body (results listed in Table 2). In summary, by utilizing the WCA scheme, the navigation sensor can image the stars and target celestial body well-exposed simultaneously within a single exposure and can reliably extract high-accuracy optical navigation measurements that satisfy the navigation demand. The night sky observation and accuracy analysis experiment validates our study conclusions.

## 7. Conclusions

In this paper, we first analyze the irradiance characteristics of a celestial body. This study aims at solving the problem that an optical navigation sensor is unable to image and expose the target celestial body and stars well-exposed simultaneously. Given that their irradiance difference is generally large, a solution that utilizes the WCA scheme is proposed. Then, celestial body edge model and star spot imaging model are established when the WCA scheme is adopted. The effect of exposure parameters on the accuracy of the star centroid estimation and edge extraction is analyzed based on the models. The AIT $T_{S}$ and well capacity $Q_{S}$ are the main factors that influence the edge detection accuracy performance of the celestial body. The edge detection error initially reaches the minimum number and then increases with the increase in $T_{S}$. An interval exists that indicates that the edge detection error is significantly small. The edge detection error initially reaches the minimum number and then increases with the increase in $Q_{S}$ when we set $T_{S}$ to an appropriate value. The well capacity is the main factor that influences the centroiding accuracy performance of a single star. The star centroiding error of a bright star decreases with the increase in $Q_{S}$. However, the centroiding error of a dim star is mainly caused by random noise, and more dim stars are recorded than bright stars in the FOV. Therefore, the overall centroiding error variation caused by the exposure parameters can be neglected. The exposure parameters are optimized to ensure that the optical navigation measurements satisfy the requirement of navigation accuracy. The optimal $Q_{S}$ and analytical solution of the optimal $T_{S}$ are obtained by conducting Monte Carlo simulation. The laboratorial and night sky experiments validate the correctness of the models, the proposed optimal exposure parameters, and other study conclusions. This study validates the feasibility of extracting attitude information and LOS vector from the sensor to the centroid of the target celestial body simultaneously by utilizing a miniaturized single FOV optical navigation sensor.

## Figures and Tables

**Table 1:** Parameters of the CMV20000 image sensor.

Parameter	Value	Parameter	Value
Active pixels	5120 × 3840	PRNU	1%
Pixel pitch	6.4 μm × 6.4 μm	DSNU	$10e^{-}⁄s$
Full well capacity, $Q_{MAX}$	$15,000e^{-}$	Read noise	$8e^{-}$
Conversion gain	$0.25DN⁄e^{-}$	Quantization bits	12
Dark current	$125e^{-}⁄s$	Quantum efficiency, $\eta_{QE}$	0.45$e^{-}⁄\mathrm{photon}$

**Table 2:** Apparent radius of the Moon under different exposure conditions.

Exposure Conditions	Apparent Radius/Pixels	Error/Pixels
Normal integration, T=0.4 ms	71.44	0.42
Normal integration, T=30 ms	75.36	4.34
WCA, $T=30\;\mathrm{ms},\;T_{S}=29.65\;\mathrm{ms},\;Q_{S}=3046e^{-}$	71.46	0.44
WCA, $T=30\;\mathrm{ms},\;T_{S}\;=\;29.67\;\mathrm{ms},\;Q_{S}=3750e^{-}$	71.36	0.34
WCA, $T=30\;\mathrm{ms},\;T_{S}\;=\;29.69\;\mathrm{ms},\;Q_{S}=4453e^{-}$	71.60	0.58
WCA, $T=30\;\mathrm{ms},\;T_{S}\;=\;29.74\;\mathrm{ms},\;Q_{S}=6093e^{-}$	72.12	1.10

**Table 3:** Centroid positions and magnitude of the identified stars.

Number	Centroid/Pixels	Magnitude	Number	Centroid/Pixels	Magnitude
1	(3439.886, 3084.843)	3.8	7	(57.533, 1493.233)	4.9
2	(2985.837, 2934.429)	3.0	8	(3045.821, 1069.692)	6.2
3	(1707.333, 2326.095)	4.4	9	(3131.244, 1017.171)	5.3
4	(2575.164, 2232.000)	5.2	10	(3624.311, 684.475)	4.8
5	(1012.179, 256.769)	5.5	11	(977.373, 675.311)	4.3
6	(3025.250, 1638.643)	5.4	12	(4056.727, 59.644)	3.1
